# Racial/ethnic- and county-specific prevalence of chronic hepatitis B and its burden in California

**DOI:** 10.1186/s41124-018-0034-7

**Published:** 2018-06-05

**Authors:** Mehlika Toy, Bin Wei, Tejpal S. Virdi, An Le, Huy Trinh, Jiayi Li, Jian Zhang, Ann W. Hsing, Samuel K. So, Mindie H. Nguyen

**Affiliations:** 1 Asian Liver Center, Department of Surgery, Stanford University School of Medicine, 780 Welch Road, CJ130D, Palo Alto, CA 94304 USA; 2 Division of Gastroenterology and Hepatology, Department of Medicine, Stanford University Medical Center, Palo Alto, CA USA; 3 San Jose Gastroenterology, San Jose, CA USA; 4 Palo Alto Medical Foundation, Mountain View, CA USA; 5 Chinese Hospital, San Francisco, CA USA; 6 Stanford Prevention Research Center, Department of Medicine and Stanford Cancer Institute, Stanford University School of Medicine, Palo Alto, CA USA

**Keywords:** HBV, Disease status, California, Burden of disease

## Abstract

**Background:**

In the United States, the highest burden of chronic hepatitis B (CHB) and CHB-related liver cancer is in the state of California, primarily in the San Francisco (SF) Bay and Los Angeles (LA) areas. The aim of this study was to estimate county-specific hepatitis B surface antigen (HBsAg) prevalence and quantify CHB cases by age, race/ethnicity, nativity, and disease activity status.

**Methods:**

Twelve counties in SF Bay Area and three large counties in LA area were included for this analysis. Race/ethnicity-specific prevalence of HBsAg for each county and the state of California as a whole, was estimated by including prevalence data from the National Health and Nutrition Examination Survey and various studies that estimated HBsAg prevalence in US and foreign-born Asian Pacific Islanders, Hispanic, and Black populations. In addition, clinical data of 2000 consecutive CHB patients (collected between 2009 and 2014) from a large clinical consortium in the SF Bay area were used to calculate the age-specific disease burden.

**Results:**

Of the 15 counties analyzed, SF had the highest HBsAg prevalence (1.78%), followed by Santa Clara (1.63%) and Alameda (1.45%). The majority of CHB cases were estimated to be in LA County (83,770), followed by Santa Clara (31,273), and Alameda (23,764). Among the CHB cases, 12.7% is active HBeAg positive, 24.2% is active HBeAg negative, and 10.6% has cirrhosis.

**Conclusion:**

This study confirms and quantifies the current burden of CHB in high endemic counties in the state of California using population-level estimates combined with clinical data including those from the community.

## Background

As a leading cause of liver disease, liver cancer, and liver transplantation, chronic hepatitis B (CHB) is an important public health problem globally [1]. In 2013, viral hepatitis took the lives of about 1.45 million people and was the seventh leading cause of death in the world, surpassing malaria and tuberculosis [1]. An estimated 850,000–2.2 million people in the United States (US) live with CHB and only 34.6% are aware of their infection [2]. The highest prevalence of CHB and CHB-related liver cancer in the U.S is in the state of California (CA), primarily in the San Francisco (SF) bay and Los Angeles (LA) areas [3]. However, only 34.6% are diagnosed [2], 33.3% of those diagnosed receive care [4], and 45% of those linked to care receive treatment if eligible according to treatment guidelines [5]. Without diagnosis, linkage to care and treatment, one in four people with CHB will die from cirrhosis, liver cancer, and/or liver failure [6]. In US regions with high proportion of immigrants, such as the SF Bay area, the vast majority of confirmed cases of CHB are Asian or Pacific Islander (API) [2]. A study from San Francisco found that of all hepatitis B virus (HBV) infection cases reported, 84% were API and of those 80% were foreign born [7]. In the U.S., the prevalence of CHB among foreign-born people is estimated to be 10 times higher than the national prevalence rate [8]. A seroprevalence survey study on Asian Americans in the SF Bay area concluded that 8.9% API were infected with CHB but 65.4% of the chronically infected adults were unaware of their infection [2]. One of the four overarching goals of the Department of Health and Human Services Action Plan for the Prevention, Care and Treatment of Viral Hepatitis is to increase the diagnosis rate of CHB from 33 to 66% by 2020 [9].

The National Health and Nutrition Examination Survey (NHANES) has been the primary data source for HBV [10] prevalence estimate in the US population. However, due to the small sample size in the survey, it is not an appropriate source for county- and state-specific prevalence estimates of CHB [11]. In addition, county-level and race/ethnicity-specific data are important for setting priorities in public health and resource allocation at the local level and since the distribution of CHB disease burden varies by race/ethnicity which itself varies by counties. Thus, to fill this gap, the aim of this study was to estimate the prevalence of HBsAg and quantify CHB active disease burden in the state of CA and certain specific counties by age, race/ethnicity, and disease activity status, using population-based data and clinical data from a large clinical and community consortium in the SF bay area.

## Methods

As a first step, we obtained age-specific population for 12 counties in the SF Bay area and 3 large counties in the LA area (Los Angeles, San Bernardino and Orange counties) as well as county-specific race/ethnicity distributions in these populations from the US census [12]. We then categorized the population according to the following four major racial/ethnic groups: White, Hispanic, Black, and API. Nativity data for Black and API populations were also obtained from the US census bureau. Approximately, 10.3% of Blacks in the US are foreign born [13], of whom 36% were born in Africa, 35% in Jamaica and Dominican Republic, 15% in Haiti, and 9% in East and South America, and 5% elsewhere [14]. Within the API population, we further divided the groups into: South Asian (23.3%), Korean (8.6%), Japanese (6.7%) and other East Asian (61.4%) [15, 16]. Secondly, we calculated age-specific API foreign-born population distribution from the US census data [17] and used these age-specific distributions for the East Asia, Korea and South Asia group. For example, the Japanese foreign born distribution was reported to be 27% in the US [15]. Next, we collected race/ethnicity- and nativity-specific HBsAg prevalence data among White [11], Hispanic [11, 18], Black [11, 19–21] and APIs [2, 11, 19, 22–24] in the US from the literature. Jung et al. reported a 0% (353 people tested) of HBsAg prevalence among 70+ year old Hispanic population, which we decided not to take and assume the same prevalence for 60+ as the 60–69 age-group (0.38%). Table 1 shows the race/ethnicity- and nativity- as well as age-specific prevalence rates, where reported, in the studies that we used as prevalence data to calculate the overall prevalence for the SF Bay area, the LA area and for the entire state of California.

**Table 1:** Race/ethnicity- and nativity-specific HBsAg prevalence data from literature review

Race/Ethnicity	Ages 0–19 Prevalence (range)	Reference	Ages > 19 Prevalence (range)	Reference
White	0.03% (0.01–0.08)	Roberts et al. [11]	0.10% (0.05–0.20)	Roberts et al. [11]
Hispanic	0.03% (0.01–0.08)	Roberts et al. [11]	20–29 0.16% (0.05–0.47)	Jung et al. [18]
			30–39 0.14% (0.04–0.42)	
			40–49 0.49% (0.24–1.00)	
			50–59 0.39% (0.21–0.72)	
			60–69 0.38% (0.10–1.37)	
			70 + ^a^ 0.38% (0.10–1.37)	
Black US-Born	0.03% (0.01–0.08)	Roberts et al. [11]	0.10% (0.05–0.20)	Roberts et al. [11]
Black Foreign-Born				
Africa	7.30% (6.50–8.00)	Ugwu et al. [20]	20–29 10.45% (9.50–11.30)	Ugwu et al. [20]
			30–39 11.20% (9.70–12.70)	
			40–49 6.99% (5.60–8.30)	
			50–59 10.86% (9.70–12.00)	
			60+ 10.86% (9.70–12.00)	
Jamaica and Dominican Rep.	0.30% (0.10–0.80)	Roberts et al. [11]	2.10% (0.70–4.00)	Din et al. [19]
Haiti	2.50% (2.10–3.00)	Tohme et al. [21]	2.50% (2.10–3.00)	Tohme et al. [21]
South & Central America	0.30% (0.10–0.80)	Roberts et al. [11]	0.60% (0.2–2.00)	Din et al. [19]
API US Born				
East Asia	0.30% (0.10–0.80)	Roberts et al. [11]	1.40% (0.65–1.90)	Din et al. [19]
Korea	0.03% (0.01–0.08)	Roberts et al. [11]	1.40% (0.65–1.90)	Din et al. [19]
Japan	0.03% (0.01–0.08)	Roberts et al. [11]	0.10% (0.05–0.20)	Roberts et al. [11]
South Asia	0.03% (0.01–0.08)	Roberts et al. [11]	1.40% (0.65–1.90)	Din et al. [19]
API Foreign Born				
East Asia	1.10% (0.90–1.90)	Shuler et al. [23]	20–29 5.40% (3.10–8.50)	Lin et al. [2]
			30–39 11.50% (8.30–15.20)	
			40–49 12.20% (9.70–15.00)	
			50–59 8.80% (7.00–10.80)	
			60–69 8.00% (5.90–10.60)	
			70–79 6.70% (4.00–10.40)	
			80+ 3.70% (1.00–9.30)	
Korea	0.30% (0.10–0.80)	Roberts et al. [11]	20–29 1.18% (0.43–2.55)	Hyun et al. [22]
			30–39 2.53% (1.61–3.77)	
			40–49 2.76% (2.00–3.70)	
			50–59 2.90% (2.23–3.69)	
			60–69 2.06% (1.37–2.96)	
			70–79 1.37% (0.59–2.68)	
			80+ 2.17% (1.17–2.77)	
Japan	0.03% (0.01–0.08)	Roberts et al. [11]	1.02%^b^ (1.01–1.02)	Tanaka et al. [24]
South Asia	0.30% (0.10–0.80)	Roberts et al. [11]	2.70% (1.60–4.00)	Din et al. [19]

Definitions of race and ethnicity according to the US Census Bureau: White- A person having origins in any of the original peoples of Europe, the Middle East, or North Africa. Black or African American- A person having origins in any of the Black racial groups of Africa. Asian- A person having origins in any of the original peoples of the Far East, South East Asia, or the Indian subcontinent including, for example, Cambodia, China, India, Japan, Korea, Malaysia, Pakistan, the Philippine Islands, Thailand and Vietnam. Native Hawaiian or other Pacific Islander- A person having origins in any of the original peoples of Hawaii, Guam, Samoa, or other Pacific Islands. Hispanic origin can be viewed as the heritage, nationality, lineage, or country of birth of the person or the person’s parents or ancestors before arriving in the US

The foreign born population includes anyone who is not a U.S citizen at birth, including those who became U.S citizens through naturalization

*API* Asian Pacific Islander, *US* United States, *HBsAg* hepatitis B surface antigen

^a^We adapted from Jung et al. and assumed a 0.38% for 70+

^b^We took a weighted average of Tanaka et al.’s HBV screening cohort ages 40–74 which we calculated to be 1.02%

In all race/ethnicity groups of ages 0–19 years, except for the Black foreign-born Africa and Haiti groups as well as the API foreign-born East Asia, we used the NHANES 2015 data. NHANES reported a prevalence of 0.03% (0.01–0.08) among 6–19 year old group, which we used for 0–19 year olds in the White, Hispanic, Black US-born, API US-born Korea, Japan and South Asia groups, as well as the Japanese foreign-born group. We used the prevalence that was 10-fold greater than the general population explanation of the NHANES study and applied it to the Black foreign-born in Jamaica and Dominican Republic, API US-born East Asian and API foreign-born Korean groups for estimating the prevalence among 0–19 year olds.

In order to quantify the age-specific “active” disease burden in relation to CHB activity and cirrhosis, we analyzed clinical baseline data such as HBV DNA and alanine aminotransferase (ALT) levels of 2000 consecutive new treatment-naïve CHB patients who presented between 2009 and 2014 at several hepatology and community gastroenterology and primary care clinics as of the SF Bay Area Consortium (SFBAC). Following the American Association for the Study of Liver Disease (AASLD) guidelines [6], disease status was defined as “active” by the following criteria: presence of cirrhosis or an elevation of ALT > 2 times the upper limit of normal (ULN) or evidence of significant histological disease plus elevated HBV DNA above 2000 IU/mL for HBeAg-negative and above 20,000 IU/mL for HBeAg-positive non-cirrhotic cases.

## Results

### Estimates of HBsAg prevalence and burden

The age-specific foreign-born distribution for API for age groups 0–19, 20–29, 30–39, 40–49, 50–59, 60–69, and 70+ were 22, 64, 83, 87, 89, 87, 90%, respectively. Combining population distribution by county, race/ethnicity and nativity with race/ethnic specific HBsAg-prevalence, we estimated that overall, all race-combined, there are 104,734 (range 70,952–153,598) CHB cases or 1.18% (range 0.80–1.73%) in the SF Bay area. Table 2 shows the estimated overall age-specific HBsAg-prevalence (all race combined) and related number of CHB cases in the SF Bay area.

**Table 2:** Estimated HBsAg prevalence and estimated number of HBsAg-positive cases in the San Francisco Bay area

Age Group (Years)	Population^a^ (2015)	HBsAg-positive cases (range)	HBsAg-prevalence (range)
0–19	2,202,500	2607 (1474–5577)	0.12% (0.07–0.25%)
20–29	1,254,437	11,106 (6113–18,053)	0.89% (0.49–1.44%)
30–39	1,311,988	24,436(16,842–33,961)	1.86% (1.28–2.59%)
40–49	1,230,512	25,627 (19,102–33,946)	2.08% (1.55–2.76%)
50–59	1,192,159	19,120 (14,218–25,231)	1.60% (1.19–2.12%)
60–69	898,217	12,940 (8709–19,866)	1.44% (0.97–2.21%)
70–79	473,888	6117 (3455–10,572)	1.29% (0.73–2.23%)
80+	310,260	2781 (1039–6393)	0.90% (0.34–2.06%)
Total	8,873,961	104,734 (70,952–153,598)	1.18% (0.80–1.73%)

^a^Total population year 2015, counties: San Francisco, Santa Clara, Alameda, San Mateo, Contra Costa, San Joaquin, Merced, Monterey, Stanislaus, Marin, Santa Cruz and San Benito

*HBsAg* hepatitis B surface antigen

As shown, the highest prevalence was amongst ages 40–49 (2.08%) with a total of 25,627 (24.5% of all cases), followed by 1.86% for those between the ages of 30 and 39. In the LA area, with a population of 15.5 million, we estimated that the overall prevalence is 0.71% (range 0.46–1.12%), with a total of 109,442 (range 71,363–172,482) with CHB. For California as a state with a population of 39.1 million, the prevalence was estimated to be 0.78% (0.51–1.21%), with a total estimated case number as 305,419 (range 200,100–475,523).

### County-, race/ethnicity-, and nativity-specific HBsAg prevalence and burden

**Fig. 1:**
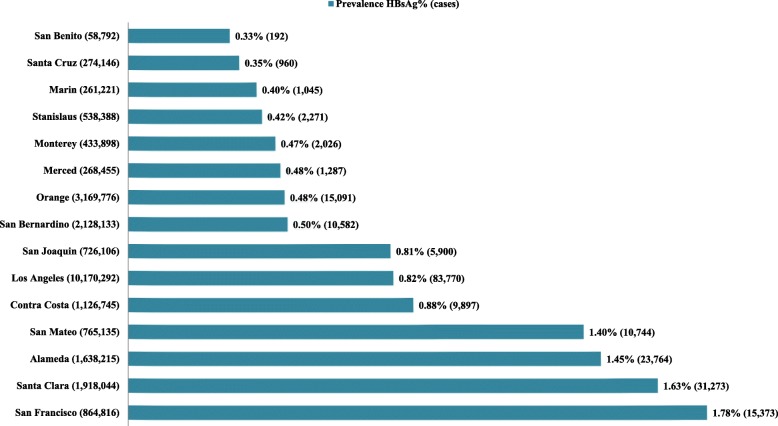
HBsAg prevalence and number of chronic hepatitis B cases in 15 counties in the State of California

Of the 15 counties analyzed, SF County had the highest HBsAg prevalence (1.78%), followed by Santa Clara (1.63%), Alameda (1.45%), San Mateo (1.40%) and Contra Costa (0.88%), as shown in Fig. 1. However, LA County (83,770), Santa Clara (31,273), Alameda (23,764) and Orange County (15,091) have high number of individuals with HBsAg due to its large population.

**Fig. 2:**
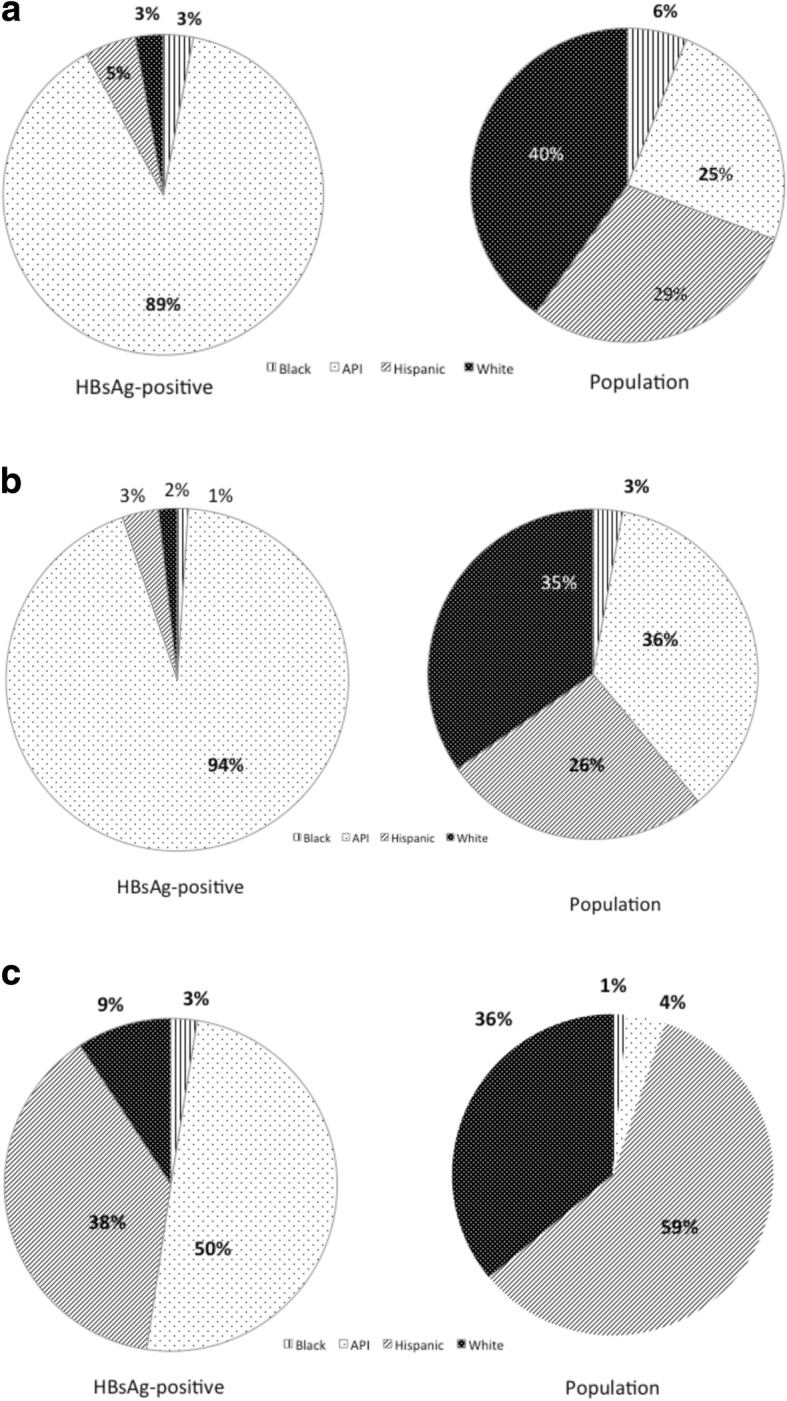
Racial and ethnic distribution among HBsAg-positive and general population in the San Francisco Bay area (**a**), Santa Clara County (**b**), and San Benito County (**c**)

Figure 2 shows the population and HBsAg distribution in the SF Bay area (Fig. 2a), Santa Clara County (Fig. 2b), and San Benito County (Fig. 2c). As shown, the SF Bay area has a 24.5% API population, but accounts for 88.5% of all CHB cases. Santa Clara County has the highest API population (36%) and they account for 94% of the HBsAg-positive cases in the county. San Benito County has the highest Hispanic population (59%), and they account for 38% of the HBsAg-positive cases in the county. In contrast, although API constitutes only 4% of the population in San Benito, they account for 50% of the HBsAg cases in this county.

In the SF Bay area, the HBsAg prevalence in API is estimated to be 4.26%. Although API comprises of 24.5% of the total population it accounts for 88.5% (92701) of the 104,734 HBsAg-positive cases. Of the HBsAg-positive cases among API cases, 4.93% were estimated to be US born and 84.0% foreign born (Table 3). Black cases (2886) made up 2.76% of total cases, with 0.39% being US born and 2.37% being foreign born.

**Table 3:** HBsAg-positive cases and its distribution by age, race/ethnicity and nativity within each age group in the total 104,734 estimated cases in the San Francisco Bay area

Age Group (Years)	API		Black		Hispanic	White	Total
	Foreign-Born	US-Born	Foreign-Born	US-Born			
0–19	889 (34.1%)	781 (30.0%)	435 (16.7%)	36 (1.38%)	204 (7.83%)	262 (10.0%)	2607
20–29	8059 (72.6%)	1497 (13.5%)	394 (3.55%)	69 (0.62%)	589 (5.30%)	498 (4.48%)	11,106
30–39	22,106 (90.5%)	777 (3.18%)	437 (1.79%)	73 (0.30%)	523 (2.14%)	520 (2.13%)	24,436
40–49	22,512 (87.8%)	540 (2.11%)	289 (1.13%)	68 (0.27%)	1724 (6.73%)	494 (1.93%)	25,627
50–59	16,399 (85.8%)	450 (2.35%)	386 (2.02%)	66 (0.35%)	1336 (6.99%)	483 (2.53%)	19,120
60–69	10,878 (84.1%)	377 (2.91%)	291 (2.25%)	50 (0.39%)	976 (7.54%)	368 (2.84%)	12,940
70–79	5077 (83.0%)	154 (2.52%)	151 (2.47%)	26 (0.43%)	515 (8.42%)	194 (3.17%)	6117
80+	2068 (74.4%)	137 (4.93%)	99 (3.56%)	17 (0.61%)	333 (12.0%)	127 (4.57%)	2781
Total	87,988 (84.0%)	4713 (4.50%)	2482 (2.37%)	405 (0.39%)	6200 (5.92%)	2946 (2.81%)	104,734 (100%)

### Active disease burden

Table 4 shows the patient characteristics of the 2000 CHB patients in the Stanford and community clinic consortium from the SF Bay area (SFBAC). Of these, 55.8% were male, and the median age at diagnosis was 43. About 22% were positive for HBeAg, with 14% non-cirrhotic active HBeAg-positive and 26% non-cirrhotic active HBeAg-negative. Approximately 9% of all cases had cirrhosis.

**Table 4:** Characteristics of the chronic hepatitis B patients from the hepatology and community gastroenterology and primary care clinics of the San Francisco Bay area consortium

Parameters	Results
Total number, N	2000
Male	1117 (55.8%)
Age	43 (18–88)
HBeAg-positive	444 (22.2%)
Active HBeAg-positive (non cirrhotic)*	272 (14%)
Active HBeAg-negative (non cirrhotic)*	510 (26%)
ALT (U/L)	39 (3–2809)
HBV DNA (log_10_ IU/mL)	4.0 (1.3–11.3)
Cirrhosis	185 (9%)

*Active is defined by an elevation of ALT > 2× upper limit of normal or evidence of significant histological disease plus elevated HBV DNA above 2000 IU/mL for HBeAg-negative, and above 20,000 IU/mL for HBeAg-positive [6]

*HBeAg* hepatitis B e antigen, *ALT* alanine aminotransferase

Applying clinical data from Table 4 to the SF Bay area CHB population, in Table 5, we show the estimated data on HBeAg, CHB activity, and cirrhosis. The number of active CHB cases, who are considered to be eligible for antiviral treatment by the AASLD guideline because they have significant viremia and evidence of on-going inflammation or hepatic fibrosis, included 14,3149 (12.6%) in the HBeAg-positive group, and 25,636 (24.5%) in the HBeAg-negative group, with 11,112 (10.6%) cirrhosis cases at baseline. In the LA area, a total of 51,952 people out of the 15.5 million population and 109,442 CHB cases are estimated to need antiviral treatment. When projected to the state of California as a whole, out of an estimated 305,419 CHB cases, 38,158 are active HBeAg-positive, 73,700 are active HBeAg-negative and 33,091 are cirrhotic. In total, an estimated 144,949 (47.5%) people with CHB in CA meet the AASLD criteria for treatment.

**Table 5:** Prevalence of chronic hepatitis B in the San Francisco Bay area by age and disease status

Age Group (Years)	Population (2015)	Inactive^a^ CHB	Active HBeAg positive	Active HBeAg negative	Cirrhosis
0–19	2,202,500	1805 (69.2%)	602 (23.1%)	201 (7.69%)	0
20–29	1,254,437	5360 (48.3%)	2744 (24.7%)	2701(24.3%)	300 (2.70%)
30–39	1,311,988	12,218 (50.0%)	4680 (19.2%)	6834 (28.0%)	704 (2.88%)
40–49	1,230,512	13,111 (51.2%)	2930 (11.4%)	7450 (29.1%)	2136 (8.33%)
50–59	1,192,159	9770 (51.1%)	1199 (6.27%)	5154 (27.0%)	2997 (15.7%)
60–69	898,217	7939 (61.4%)	438 (3.38%)	1813 (14.0%)	2751 (21.3%)
70–79	473,888	3186 (52.1%)	382 (6.25%)	1020 (16.7%)	1529 (25.0%)
80+	310,260	1448 (52.1%)	174 (6.25%)	463 (16.7%)	695 (25.0%)
Total	8,873,961	54,837 (52.4%)	13,149 (12.6%)	25,636 (24.5%)	11,112 (10.6%)

^a^Inactive CHB are those who are HBsAg positive with low HBV DNA or high HBV DNA but normal alanine aminotransferase or ALT levels

*HBeAg* hepatitis B e antigen

## Discussion

This study confirms and quantifies the current burden of CHB in high endemic counties in the state of CA using population-level estimates combined with real-world clinical data including those from the community. We estimated the overall all race-combined HBsAg-prevalence of the SF Bay area, LA area, and the state of CA to be 1.18, 0.71 and 0.78%, respectively. The national US HBsAg prevalence estimated by NHANES is only 0.3% [11]. According to our estimates, SF Bay area prevalence surpasses the 0.3% by about 4-fold. The prevalence is highest in the SF (1.78%), Santa Clara (1.63%), and Alameda (1.45%) counties. However, the burden based number of patients is highest in the LA (83,770), Santa Clara (31,273) and Alameda (23,764) counties, due to the large population, especially APIs, in these counties. As expected, a significant portion of the burden is among the API population, with an estimated 4.26% HBsAg-prevalence. This rate is higher than the 3.1% reported by the NHANES 2015 study as the true prevalence of CHB infections for Asians [11], including both immigrants and naturalized citizens. Our estimate is comparable to the 3.8% prevalence reported by Levy et al. among men aged 18–35 years residing in low-income neighborhoods in Northern California [25]. Although the Centers for Disease Control and Prevention (CDC) estimates that Asians account for 50% of all CHB cases in the US [26], our study estimated that in CA, especially in the SF Bay area, APIs make up 88.5% of all the CHB cases. Particularly relevant is the high proportion of total CHB cases among the API foreign-born group that account for 84.0% of all the cases in the SF Bay area. It is estimated that the overall burden of CHB in the U.S. will continue to increase with ongoing immigration from countries with intermediate (2–8%) to high (> 8%) HBV prevalence [8]. For the foreign born population, we tried to use prevalence data from the US immigrant studies where available rather than the prevalence in their home country.

It is noteworthy that although currently both prevalence of HBV infection and incidence of hepatocellular carcinoma (HCC) due to HBV are high in the API population, data from SEER registries have shown a decline in HCC incidence among Asians, while rates in other racial/ethnic groups will continue to rise [27, 28]. In fact, in a national forecast study using SEER national data, by 2030, Hispanics will have the highest HCC in the country, while Asians will have the lowest HCC rates [28]. It is important to note that Hispanic is the largest racial/ethnic population in the state of California, accounting for 39% of the total CA population [29]. Relative to Asians, Hispanics tend to have lower CHB infection but higher HCV infection. Although our HBsAg-prevalence estimate of the Hispanic population in the SF Bay area is only 5% of the total, in counties that have large Hispanic populations such as San Benito and Merced, the Hispanic population makes up to 38% of all CHB cases. During the 2006–2010 period, among individuals aged 50–64 years, Hispanics experienced higher HCC incidence and mortality rates than Asians and Whites [30]. Few studies have investigated risk factors for HCC in Hispanics and the reasons for the rising trend in this ethnic group, although one study did show that HBV infection is one of the risk factors for HCC in Hispanic [30].

Although we analyzed 62% of the total population of California, it is unclear whether we can generalize the data from SF and LA reported here to the entire state of California, as SF and LA cover counties that are more densely populated and more diverse, and the remainder 43 counties in the state are quite different. However, our clinical data, including HBeAg status, among CHB patients from the university liver and community gastroenterology and primary care clinics were comparable to those reported by a North American multicenter study [31], suggesting some utility of generalizing our estimates to the state of CA. As there are many immigrants in California from the Middle East, which historically had a higher HBV prevalence than Western Europe or the European settlers of North America, the NHANES study we used to estimate the prevalence among whites does not differentiate among the white population which is likely a limitation in our study.

Given the high burden of CHB and HCC in the SF Bay area and California, targeted prevention and control efforts are needed to minimize the burden in these communities. A recent CDC-funded study showed that in cities with large populations of Asia- and Africa-born immigrants, community-based and refugee clinic-based HBV testing initiatives can identify substantial numbers of individuals with CHB [32], and stresses the fact that culturally and linguistically specific approaches were necessary in all phases of the initiatives. Our data and estimates can be used for resource allocation planning to target specific geographic areas and subpopulations, race/ethnicities for effective interventions, such as screening and linkage to care with appropriate therapies, to minimize the burden of CHB and its clinical consequences, such as liver cirrhosis, and liver cancer. These interventions are urgently needed as currently most of the CHB patients (70%) in the US are not aware of their HBV infection and there are also large gaps at various levels of the cascade of care for those with known CHB [5, 33–35]. Importantly, improved linkage to care can identify patients who may benefit from antiviral therapy which has been shown in both randomized control studies as well as large cohort studies in the US to substantially reduce HCC risk in CHB patients [36, 37].

## Conclusion

In summary, our data suggest that there are approximately 305,000 persons living with CHB in the State of CA, and about half of these (47.5%, 150,000) have cirrhosis and/or meeting AASLD guideline criteria for antiviral therapy. We also found significant variation in the racial/ethnic make-up of the CHB population in different regions and counties of the State, highlighting the need for region/county-specific approach for public health efforts targeting CHB patients. Our methodology can be applied in other states or regions to estimate specific county, age, racial/ethnic prevalence and the overall and distribution active/advanced disease burden that would be crucial in the planning of local public health efforts in those areas.

## Abbreviations

AASLD: American association for the study of liver disease
ALT: Alanine aminotransferase
API: Asian or pacific islander
CA: California
CDC: Centers for Disease Control and Prevention
CHB: Chronic hepatitis B
DNA: Deoxyribonucleic acid
HBsAg: Hepatitis B surface antigen
HCC: Hepatocellular carcinoma
LA: Los Angeles
NHANES: National Health and Nutrition Examination Survey
SF: San Francisco
SFBAC: San Francisco Bay Area Consortium
ULN: Upper limit of normal
US: United States
